# Distance traveled for Medicaid-covered abortion care in California

**DOI:** 10.1186/s12913-017-2241-0

**Published:** 2017-04-19

**Authors:** Nicole E. Johns, Diana Greene Foster, Ushma D. Upadhyay

**Affiliations:** Advancing New Standards in Reproductive Health (ANSIRH), Bixby Center for Global Reproductive Health, Department of Obstetrics, Gynecology and Reproductive Sciences, University of California, San Francisco, 1330 Broadway, Suite 1100, Oakland, CA 94612 USA

**Keywords:** Abortion, Medicaid, Travel distance, Rural, Access

## Abstract

**Background:**

Access to abortion care in the United States is limited by the availability of abortion providers and their geographic distribution. We aimed to assess how far women travel for Medicaid-funded abortion in California and identify disparities in access to abortion care.

**Methods:**

We obtained data on all abortions reimbursed by the fee-for-service California state Medicaid program (Medi-Cal) in 2011 and 2012 and examined distance traveled to obtain abortion care by several demographic and abortion-related factors. Mixed-effects multivariable logistic regression models were constructed to examine factors associated with traveling 50 miles or more. County-level t-tests and linear regressions were conducted to examine the effects of a Medi-Cal abortion provider in a county on overall and urban/rural differences in utilization.

**Results:**

11.9% (95% CI: 11.5–12.2%) of women traveled 50 miles or more. Women obtaining second trimester or later abortions (21.7%), women obtaining abortions at hospitals (19.9%), and rural women (51.0%) were most likely to travel 50 miles or more. Across the state, 28 counties, home to 10% of eligible women, did not have a facility routinely providing Medi-Cal-covered abortions.

**Conclusions:**

Efforts are needed to expand the number of abortion providers that accept Medi-Cal. This could be accomplished by increasing Medi-Cal reimbursement rates, increasing the types of providers who can provide abortions, and expanding the use of telemedicine. If national trends in declining unintended pregnancy and abortion rates continue, careful attention should be paid to ensure that reduced demand does not lead to greater disparities in geographic and financial access to abortion care by ensuring that providers accepting Medicaid payment are available and widely distributed.

**Electronic supplementary material:**

The online version of this article (doi:10.1186/s12913-017-2241-0) contains supplementary material, which is available to authorized users.

## Background

Access to abortion care in the United States (US) is limited by the availability of abortion providers—the number of providers, their geographic distribution, and their willingness to accept insurance. The number of abortion providers in the country has declined in recent years; the most recent census of abortion providers estimated a total of 1720 in the US in 2011, down 4% in just three years from 1793 in 2008 and down 5% from 1819 in 2000 [1]. The geographic distribution of providers is not uniform; most providers are concentrated in major cities and not easily accessed by would-be patients in more rural areas. While among all US counties in 2011, 89% had no abortion provider (and were home to 38% of US women aged 15–44), rural counties were less likely to have a provider: 97% of rural counties had no provider compared to 69% of urban counties [1, 2]. In California, 45% of counties (home to 5% of CA women 15–44) had no abortion provider [1].

Existing studies have examined geographic accessibility to abortion, though to our knowledge no study has examined distances traveled specifically by those using Medicaid funds, or more broadly, by low-income women who may be most burdened by additional travel costs and time. Average distance traveled for abortion has been estimated at the national and regional levels for the general population of abortion patients in the United States [3], and distance estimates exist at the state level for Louisiana [4], New York [5] and Texas [6, 7]. Studies have also used estimates by clinic managers and other key informants at facilities to calculate the proportion of patients traveling distances greater than 50 miles to reach the abortion facility [8, 9]. Estimates of the percent of women traveling greater than 50 miles have changed little over time; in 2005 27% of abortion patients traveled >50 miles, in 2001, 1997, and 1993 an estimated 24% traveled >50 miles [8–10]. A nationally representative survey of abortion patients in 2008 estimated that women traveled an average of 30 miles for abortion services, with a median of 15 miles and that 67% traveled less than 25 miles, 16% 25–29 miles, 11% 50–100 miles and 6% more than 100 miles for care [3]. Controlling for other factors, this study found that rural women and women obtaining second trimester abortions were more likely to travel greater distances, while women of color were less likely to travel long distances than non-Hispanic white women.

Distance traveled to abortion has been studied in several other countries as well, and these studies suggest that rural and minority groups travel furthest to obtain abortion care. Studies in Canada found, as in the US, geographic disparities in abortion access due to clustering of provision in urban centers [11]. One national study found that 18% of women traveled more than 100 km (62 miles) to reach an abortion clinic and that younger woman and Aboriginal women traveled significantly further for services than older and white women [12]. A review of studies examining barriers to abortion access in Australia found that interstate travel for abortion was common, rural access to abortion was limited, and that greater travel distances were associated with greater costs [13]. In New Zealand, a study of access to first trimester abortion found that women in regions without an abortion provider had to travel on average 137 miles each way to reach abortion services, and that regions without a provider had higher than average native (Maori) populations [14].

Not all abortion providers offer all types of abortion care; compared to first trimester abortion providers, second trimester providers are relatively scarce. Almost all (95%) abortion providers offer abortion care at 8 weeks since last menstrual period (LMP); however, this drops to 61% offering care past 12 weeks LMP, and 16% offering care past 24 weeks LMP [15]. Consequently, women who do not access a provider in their first trimester may find it more difficult to find a provider in the second trimester, and increasingly so as time passes [16–18]. Previous studies have shown that women presenting for abortions in the second trimester travel longer distances than women in the first trimester [19, 20]. This is clinically problematic because increased gestation is associated with greater risk of morbidity [21, 22] and mortality [23, 24]. For example, the rate of major adverse events after a first trimester abortion is 0.16%, and after second trimester or later abortions is 0.41% [25]. While these rates are already extremely low, they could be further reduced if women seeking abortion could obtain care as early as desired. The distance a patient must travel to reach a provider may affect her ability to receive timely care, or any care at all.

Currently, public funding for abortion care is available in only 17 states [26]. California is one of these states; California’s Medicaid program, Medi-Cal, covers about half of California abortions [1, 27]. However, public funding for abortion does not guarantee that publically funded abortions will be accessible. California-based studies have found that difficulty getting Medi-Cal to pay for abortion contributes to delays in obtaining abortion, pushing women into the second trimester. These difficulties include women’s lack of knowledge about available coverage, difficulty negotiating the Medi-Cal application process and difficulty locating an abortion provider that accepts Medi-Cal payment [16, 18]. Given these barriers, it is likely that many women who are eligible for Medi-Cal-coverage for their abortions will choose to instead pay out-of-pocket if they can. In a multi-state study of barriers to Medicaid acceptance for abortion, providers cited low Medicaid abortion reimbursement rates as the primary barrier to accepting Medicaid [28]. Complex billing procedures and slow reimbursement times were also frequently mentioned. A 2006 examination of Medi-Cal acceptance among abortion providers publically advertising in Yellow Pages found that only 53% accepted Medi-Cal through the first trimester, 20% accepted Medi-Cal up to 20 weeks gestation, and 4% accepted Medi-Cal past 21 weeks [29]. Though surveys identified 512 total abortion providing facilities in California in 2011 [2], it is not known how many of these facilities accepted Medi-Cal payment.

A simple count of abortion providers in each state does not distinguish between types of providers in terms of gestational limits, eligible patients, or payment types accepted. Hospitals are less likely to accept patients other than for medical indications or high-risk conditions which could not be managed in typical outpatient settings, and obstetrician/gynecologist or family physician private practice offices are unlikely to accept patients outside their own established patient base [8]. Practices differ in gestational limits, types of procedures, availability and open hours, costs and payment types accepted, and protocols, all of which may affect which abortion provider a woman is actually able to access.

Though distance to care is known to affect abortion access, a comparison of abortion rates by geography may be complicated by cultural differences in rural populations, such as fertility preferences, attitudes towards abortion, and abortion stigma, which may cause differences in the utilization of abortion beyond those caused by increased distance alone [30]. One of the earliest studies of distance traveled for abortion post Roe v. Wade found that the greater the distance from an abortion clinic, the lower the abortion rate, but acknowledged that lower abortion rates may be a function of women in more rural areas far from abortion clinics preferring not to terminate a pregnancy as well as a lack of knowledge of the availability and location of abortion providers [31]. Rural populations are less likely to support abortion under a range of circumstances than their urban counterparts [32] and studies have shown higher fertility rates paired with lower abortion rates among rural women compared to urban women in specific contexts [33]. Although rural women are underrepresented among abortion patients [3], the reasons for this are not well understood.

In this study we examine the distances women travel for Medi-Cal-covered abortion care in California, the factors associated with traveling longer distances, and the facilities offering abortion care to Medi-Cal beneficiaries using a unique dataset on abortions covered by California’s state Medicaid program in 2011 and 2012.

## Methods

We conducted a retrospective observational cohort study of abortion claims data in the California Medicaid program (Medi-Cal). We obtained data on all abortions covered by the fee-for-service (FFS) Medi-Cal program in 2011 and 2012 from California’s Department of Health Care Services (DHCS). The study was approved by the institutional review boards of the University of California, San Francisco and the California Health and Human Services Agency.

We obtained aggregate data on the number of Medi-Cal enrollee women of reproductive age by county to examine the geographic distribution of providers compared to the county-level geographic distribution of eligible women and to calculate abortion rates. We also obtained aggregate data on births to the Medi-Cal FFS population for 2012 (2011 data were not available) to calculate abortion ratios.

Medi-Cal is administered on a fee-for-service or managed care arrangement, with roughly equal numbers of women enrolled in each at the time of the study. Pregnant women have four options for health care coverage under Medi-Cal: Full-coverage Medi-Cal, Pregnancy-related Medi-Cal (covering pregnancy-related healthcare only), Presumptive Eligibility for Pregnant Women (temporary pregnancy-only coverage), and Medi-Cal Access Program (for those women whose incomes are too high to qualify for Medi-Cal, coverage of all healthcare during and shortly after pregnancy for a low-cost premium). While the California Department of Health Care Services considers the Medi-Cal Access Program and Presumptive Eligibility for Pregnant Women fee-for-service programs, only the traditional fee-for-service billing records (both full-coverage and pregnancy-only) contain complete information for care provided to the beneficiary; therefore, we requested data only for those beneficiaries with traditional fee-for-service coverage. These claims represent approximately one quarter of all Medi-Cal covered abortions [27].

### Measures

For each Medi-Cal beneficiary, the dataset included an encrypted ID number, date of birth, city, state, zip code, longitude and latitude of the beneficiary residence, race, date(s) of service, diagnoses (International Classification of Diseases, 9th Revision [ICD-9] codes), and procedures or treatments. For each procedure, the dataset also included the provider number, the address, city, state, zip code where provider is registered, the facility type, and amount paid per individual treatment. For additional details on data preparation, see Additional File 1.

The primary outcome of interest was the distance that each beneficiary traveled to obtain her abortion. We used TRAVELTIME3, a STATA module that uses a Google Maps application programming interface (API) to calculate distance traveled and travel time via road to the provider for each beneficiary [34, 35]. We categorized the distance variable into four groups: <25 miles, 25–49 miles, 50–99 miles, and 100+ miles. We also dichotomized distances and times to less than 50 miles or 50 miles or more, and examined which factors were associated with greater distances traveled to seek abortion care.

We also quantified the number of abortion providing facilities. We first estimated the number of facilities reimbursed for at least one abortion over the two years. We also estimated the number of facilities reimbursed for at least 50 abortions over the two years as an indicator of facilities that routinely accepted Medi-Cal for abortion. Among those facilities providing at least 50 total abortions, we also calculated the proportion providing at least one medication abortion, first trimester aspiration abortion, and second trimester or later abortion.

We calculated abortion rates and abortion ratios for each of the 58 counties. Abortion rates are the number of abortions per 1000 women of reproductive age and abortion ratios are the number of abortions per 1000 live births.

### Statistical analysis

First we present the characteristics of the sample and estimated median distance traveled by age, race, urban/rural residence, procedure type (medication abortion, first trimester aspiration, second trimester or later abortion), and source of care. We then present the proportion of women traveling <25 miles, 25–49 miles, 50–99 miles, and 100+ miles for each of these characteristics. We then built a multivariable mixed-effects logistic regression model to examine the factors associated with traveling 50 miles or more to obtain an abortion, accounting for clustering of multiple abortions by the same woman using random effects specifications. We did not include the rural/urban indicator in the model because it was highly correlated with the outcome, distance traveled. In these analyses, the abortion is the unit of analysis.

Next we estimated the median reimbursement for all related services on the day(s) of abortion by procedure type. Finally, we examined the distribution of Medi-Cal FFS abortion-providing facilities by county. We counted facilities reimbursed for at least one and at least 50 abortions and classified facilities by county. We tested for associations between presence of, number of, and distance traveled to facilities and Medi-Cal FFS abortion rates and ratios at the county level. We used t-tests for presence of facility comparisons and linear regression to examine number of facilities and median distances. We examined the percentage of facilities performing medication abortion, first trimester aspiration abortion, and second trimester or later abortion. To test the hypothesis of whether factors beyond long distances from care, such as fertility preferences or attitudes towards abortion, cause differences in urban/rural abortion access, we also developed several models to examine abortion rates among rural and urban women. Using census data on percent of county population residing in a rural location, we examined whether median distance traveled for abortion, abortion rates, or abortion ratios differed by the percent of county living rurally, using linear regression [36]. Statistical significance was set at *p* < 0.05 for all comparisons and adjusted odds ratios (AORs) and 95% confidence intervals are reported. All statistical analyses were performed using STATA 14.1. In accordance with DHCS Public Reporting Guidelines, cells smaller than *n* = 11 were suppressed.

## Results

The dataset contained 39,747 abortions obtained by 36,720 beneficiaries of the Medi-Cal FFS program in 2011 and 2012. Among these abortions, 89% had a full and valid address available for both beneficiary and provider. Of those missing an address (*n* = 4316), 99% were missing the beneficiary address; the remaining 1% (*n* = 31) had addresses out of state or without identifiable driving distance. Of those missing distance, 98% were under the age of 21 and covered by a specific minor consent program; the Medi-Cal program suppresses data for these participants. For this analysis, we excluded those missing distance. Our final analytic sample thus included 35,431 abortions to 32,582 women.

The median age of the population was 26; the largest proportion of the population was Hispanic/Latina (50%), followed by white (25%), black (13%) and Asian (5%) (see Table 1). Of all abortions, 28% were medication abortions, 56% were first trimester aspiration abortions, and 16% were second trimester or later abortions. The majority took place in outpatient clinics (56%), followed by physician’s offices or groups (36%) and hospitals (7%).

**Table 1:** Characteristics of Women Obtaining Abortions Covered by Medi-Cal, 2011–2012

	n	%
Total	35431	100
Sociodemographics		
Age, years^a^		
17 or younger	1154	3.3
18–24	14272	40.3
25–34	15561	43.9
35 or older	4438	12.5
Race/ethnicity		
Non-Hispanic white	8951	25.3
Non-Hispanic black	4432	12.5
Hispanic/Latina	17768	50.1
Asian	1934	5.5
Other	1269	3.6
Declined to state/missing	1077	3.0
Residence		
Urban	31734	89.6
Rural	3697	10.4
Characteristics of abortion		
Abortion procedure		
Medication abortion	10037	28.3
1^st^ Trimester aspiration	19819	55.9
2^nd^ Trimester or later	5575	15.7
Source of care		
Hospital	2528	7.1
Outpatient clinic	20015	56.5
Physician’s office/Physician’s g﻿roup	12888	36.4
Data year		
2011	19215	54.2
2012	16216	45.8
Distance traveled for care		
< 25 miles	26683	75.2
25–49 miles	4540	12.8
50–99 miles	2740	7.7
100+ miles	1468	4.1

^a^Data on women missing age are not presented due to small numbers

Among all women, the mean distance traveled was 23.5 miles (95% CI: 23.1–23.9), with a range of 0.02 to 661 miles and a median distance traveled of 10.5 miles (95% CI: 10.3–10.7). 11.9% (95% CI: 11.5%–12.2%) traveled 50 miles or more to obtain an abortion and 4.1% (95% CI: 3.9%–4.4%) traveled 100 miles or more (See Table 2). Most likely to travel 50 miles or more and 100 miles or more were women obtaining second trimester or later abortions (21.7% 50+, 9.4% 100+), women obtaining abortions at hospitals (19.9% 50+, 9.9% 100+), and rural women (51.0% 50+, 19.5% 100+). Median distance traveled by abortion type was 8.7 miles for medication abortion (95% CI: 8.4–9.0), 10.2 miles for first trimester aspiration abortion (95% CI: 10.0–10.3), and 15.8 miles for second trimester or later abortion (95% CI: 15.4–16.4).

**Table 2:** Proportion of Abortions by Distance Category, Medi-Cal 2011–2012

	n	%0–24 Miles	%25–49 Miles	%50–99 Miles	%100+ Miles	Median distance Traveled (miles)	Chi-squared, K-sample equality of medians test
Total (N)	35431	26683	4540	2740	1468	10.5	
Total (%)		75.3	12.8	7.7	4.1		
Sociodemographics							
Age, years^a^							<0.001
17 or younger	1154	68.7	16.3	9.4	5.6	11.9	
18–24	14272	74.2	13.1	8.3	4.4	11.2	
25–34	15561	75.9	12.6	7.5	4.0	10.2	
35 or older	4438	78.8	11.8	6.1	3.2	9.3	
Race/ethnicity							<0.001
Non-Hispanic white	8951	60.4	19.3	13.9	6.4	17.0	
Non-Hispanic black	4432	79.9	9.6	8.0	2.5	10.3	
Hispanic/Latina	17768	80.5	10.8	5.1	3.5	8.9	
Asian	1934	82.4	10.8	4.1	2.8	9.4	
Other	1269	75.6	11.0	8.0	5.5	9.4	
Declined to state/missing	1077	81.1	11.3	4.8	2.8	11.0	
Residence							<0.001
Urban	31734	82.0	10.7	5.0	2.3	9.2	
Rural	3697	19.1	30.9	31.5	19.5	50.7	
Characteristics of abortion							
Abortion procedure							<0.001
Medication abortion	10037	79.5	12.1	5.9	2.5	8.7	
1^st^ Trimester aspiration	19819	76.6	12.6	7.4	3.5	10.2	
2^nd^ Trimester or later	5575	63.2	15.0	12.3	9.4	15.8	
Source of care							<0.001
Hospital	2528	68.8	11.3	10.0	9.9	9.6	
Outpatient clinic	20015	75.2	14.0	7.5	3.3	11.0	
Physician’s office/Physician’s g﻿roup	12888	76.8	11.3	7.6	4.3	9.9	
Data year							0.184
2011	19215	75.3	12.7	7.9	4.1	10.6	
2012	16216	75.4	13.0	7.5	4.2	10.4	

^a^Data on missing age not presented due to small numbers

In a multivariable model, several factors were associated with likelihood of traveling 50 miles or more for Medi-Cal FFS funded abortion care (see Table 3). Adolescents and young women under 18 were significantly less likely to travel 50 miles or more compared to women ages 18–24 (AOR = 0.03, 95% CI 0.003–0.37, *p* < 0.01). Hispanic and Asian women were significantly less likely to travel 50 miles or more compared to white women (*p* < 0.05). Compared to women obtaining a first trimester aspiration abortion, women obtaining a medication abortion had lower odds of traveling 50 miles or more (*p* < 0.001) and women obtaining a second trimester or later abortion had over 8 times the odds of traveling 50 miles or more (*p* < 0.001). Compared to women obtaining an abortion in an outpatient clinic, women going to a physician’s office had 2.8 times higher odds of traveling 50 miles or more (*p* < 0.001) and those going to a hospital had 6.9 times higher odds of travel 50 miles or more (*p* < 0.001). There was no difference in odds of traveling 50 miles or more by year.

**Table 3:** Odds of Traveling 50 Miles or More for Abortion Covered by Medi-Cal, 2011–2012 (*N* = 35425^a^)

Characteristic	Traveled 50+ miles Adjusted Odds Ratio	95% CI
Sociodemographics		
Age, years^a^		
17 or younger	0.03**	(0.003,0.37)
18–24	Ref	Ref
25–34	0.58	(0.28,1.19)
35 or older	0.33	(0.08,1.32)
Race/Ethnicity		
Non-Hispanic white	Ref	Ref
Non-Hispanic black	0.42	(0.14,1.32)
Hispanic/Latina	0.37*	(0.17,0.83)
Asian	0.02***	(0.002,0.19)
Other	0.22	(0.04,1.17)
Declined to state/missing	0.09*	(0.01,0.71)
Characteristics of abortion		
Abortion procedure		
Medication abortion	0.18***	(0.08,0.40)
1st Trimester aspiration	Ref	Ref
2^nd^ Trimester or later	8.81***	(4.41,17.61)
Source of care		
Hospital	6.88***	(2.53,18.70)
Outpatient clinic	Ref	Ref
Physician’s office/Physician’s g﻿roup	2.80**	(1.42,5.54)
Data year		
2011	Ref	Ref
2012	2.84***	(1.66,4.85)

^a^Abortions among women missing age (*n* = 6) were dropped from this analysis

*p < 0.05, **p < 0.01, ***p < 0.001

Median reimbursement rates for all costs on the day(s) of the abortion differed by abortion procedure. Facilities were reimbursed a median of $475 for medication abortion, $405 for first trimester aspiration abortion and $499 for second trimester or later procedures.

We identified 287 unique locations which were reimbursed by Medi-Cal FFS for at least one abortion. Of these, 65 facilities were reimbursed for only one abortion over the two years and 115 facilities were reimbursed for 50 or more abortions. Of the 58 counties in California, 30 (52% of counties) had facilities that were reimbursed by Medi-Cal FFS for at least 50 abortions; these counties were home to 90% of eligible enrollees (See Table 4). Among facilities providing at least 50 abortions, 69% (79) were reimbursed for first trimester aspiration abortions, 86% (99) were reimbursed for medication abortions, and 55% (63) were reimbursed for second trimester or later abortions. No counties with fewer than 50 abortions had an abortion provider; the 50 abortion cutoff therefore did not exclude low-volume providers meeting demand in counties with few abortions.

**Table 4:** Facilities providing 50+ Medi-Cal FFS abortions, median distance, abortion and birth rates, by county 2011–2012

County	Number of facilities providing at least 50 abortions	Median distance traveled by women residing in county (miles)	Abortion Rate (Abortions per 1000 Medi-Cal FFS female enrollees 15–49)	Birth Rate (Medi-Cal FFS funded births per 1000 Medi-Cal FFS female enrollees 15–49, 2012 only^b^)
Overall	115	11	9.4	72.1
Los Angeles	29	8	7.4	68.6
San Diego	10	10	13.8	84.6
Santa Clara	9	7	12.0	66.5
Orange	7	8	5.2	106.2
Sacramento	6	9	9.0	54.0
Riverside	5	16	7.3	87.7
San Francisco	5	4	21.0	56.6
Contra Costa	4	18	6.0	60.7
San Bernardino	4	20	5.8	68.8
San Joaquin	4	7	5.8	60.3
Alameda	3	13	10.3	58.1
Santa Barbara	3	4	16.4	98.4
Butte	2	19	12.3	59.1
Fresno	2	7	5.0	61.6
Monterey	2	16	10.4	95.0
San Luis Obispo	2	17	22.0	97.7
Solano	2	10	4.2	59.3
Sonoma	2	7	10.2	106.7
Stanislaus	2	15	6.8	64.8
Ventura	2	10	14.5	112.3
Humboldt	1	14	11.4	70.1
Kern	1	13	4.9	63.8
Madera	1	23	5.0	64.9
Mendocino	1	56	10.7	80.5
Napa	1	14	3.4	100.4
Placer	1	20	14.7	55.7
San Benito	1	16	14.3	73.7
Santa Cruz	1	6	5.9	105.7
Shasta	1	7	17.0	62.3
Sutter	1	44	10.2	66.8
Alpine	0	-^a^	-^a^	-^a^
Amador	0	47	11.8	60.4
Calaveras	0	53	9.2	49.9
Colusa	0	60	7.7	96.3
Del Norte	0	88	6.0	55.4
El Dorado	0	44	13.5	62.1
Glenn	0	24	9.5	75.4
Imperial	0	119	7.3	74.1
Inyo	0	217	6.1	71.9
Kings	0	36	3.0	60.1
Lake	0	62	11.2	56.8
Lassen	0	104	6.4	60.6
Marin	0	22	8.8	80.4
Mariposa	0	67	14.2	63.1
Merced	0	44	4.2	80.1
Modoc	0	157	6.7	22.6
Mono	0	311	7.6	95.9
Nevada	0	55	16.7	57.8
Plumas	0	89	8.7	50.7
San Mateo	0	12	9.9	85.4
Sierra	0	118	9.7	-^a^
Siskiyou	0	98	7.3	59.7
Tehama	0	34	11.0	68.7
Trinity	0	64	9.0	57.3
Tulare	0	46	3.0	63.3
Tuolumne	0	53	11.4	57.8
Yolo	0	23	10.8	91.0

^a^Suppressed due to small numbers

^b^Birth data was only available for 2012

Median distance traveled for abortion care differed by county, ranging from 4 to 311 miles. Counties with a facility providing at least 50 abortions had significantly lower median distance traveled for care by women in those counties compared to counties without a facility providing abortion (15 miles vs 77 miles, *p* < 0.001).

**Fig. 1:**
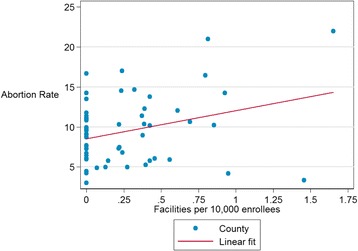
County Medi-Cal abortion rates vs. number facilities proving 50+ abortions per 10,000 female reproductive-aged enrollees

To examine whether facility availability impacted abortion utilization, we examined the relationship between facilities and abortions at the county level. We used both abortion rates and ratios because each measure reflects different contexts; abortion rates (abortions per 1000 female Medi-Cal FFS enrollees of reproductive age) relate abortions to the population ‘at-risk’, while abortion ratios (abortions per 1000 Medi-Cal FFS paid births) relate abortions to a measure of fertility. T-tests comparing county level abortion rates and ratios between counties with and without a facility providing 50 or more Medi-Cal FFS abortions found no significant associations (*p* = 0.29 & *p* = 0.79, respectively). A linear regression of county level abortion *ratios* by number of facilities (providing 50 or more abortions) per 10,000 enrollees also found no significant association (*p* = 0.51), however, the abortion *rate* was significantly positively associated with the number of facilities per 10,000 enrollees (*p* = 0.02) (see Fig. 1).

At the individual level, rural women traveled longer distances than their urban counterparts (see Table 2). We also found evidence of this at the county level; using census data on percent of population living in urban vs rural zip codes by county, we found that the percentage of a county population that is rural was positively and significantly associated with median distance traveled by women in that county in a linear regression model (*p* < 0.001). We also used linear regression to examine whether the abortion rate or ratio differed by urban/rural status, regressing percentage of the county population that is rural on abortion rate and on abortion ratio. Neither abortion rate nor ratio significantly differed by percentage of the county population that is rural (*p* = 0.91 & *p* = 0.12 respectively).

## Discussion

Despite living in California, a state with liberal abortion laws and relatively good access to abortion [1], many low-income women with Medicaid travel long distances for publicly funded abortion care. In this study, 12% of women traveled 50 miles or more to obtain a publicly funded abortion. Distances were highest among rural women, about half of whom traveled 50 miles or more. Less than a quarter of the abortion providers in the state provided significant numbers of abortions for the Medicaid program [1]. Only about half of California’s counties had facilities that regularly provided abortion care and accepted Medi-Cal as a form of payment.

The geographic distribution of abortion providers is influenced by a complex set of factors. These factors include community-level fertility preferences and attitudes about abortion, state-level restrictions, unintended pregnancy rates, population density, and the availability of trained abortion providers. Abortion care facilities are concentrated in urban areas, where populations are higher and thus a greater number of women seek abortion. Abortion providers may also concentrate in urban areas due to higher abortion stigma in more rural settings. A national study that found that obstetrician–gynecologists with rural mailing addresses were significantly less likely to perform abortions (6.5%) than their urban counterparts (17.0%) [37]. Maintaining an abortion practice can be difficult and even dangerous in a hostile and isolating environment [15, 38, 39].

Our finding that rural women travel further for care is consistent with previous research in rural women’s health. Rural women are known to experience poorer health outcomes and have less access to health care than urban women [40]. However, the county level findings demonstrate that the abortion rate does not differ by the percent of women living in rural areas. That is, rural women have similar rates of publicly funded abortion as urban women and thus need comparable access to abortion care. This finding conflicts with the earliest study on this topic that suggested that rural women access abortion at lower rates than urban women, and also runs counter to the hypothesis that social and cultural factors cause significant differences in abortion utilization by rural and urban women [31]. The distribution of facilities does not reflect this similar utilization, however.

California has few state restrictions that would result in the closure of abortion facilities [41, 42] compared to many other states, particularly in the South and Midwest [43]. However, as unintended pregnancy and abortion rates decline, [44] the number of abortion providers are also declining [45]. In California, 12 clinics have closed since 2011, [46] likely due in part to decreased demand. The closure of one facility in a city with many clinics may not have widespread impact on access, but the closure of a facility in a more rural setting may dramatically impact how far women in the surrounding areas have to travel for care, if they are able to reach a provider at all. Even a small reduction in the number of providers has the potential to reduce access to care.

We found that black, Latina and Asian women traveled shorter distances than white women, which is also consistent with national findings [3]. This finding could be because women of color may be concentrated in urban centers but could also be in part because women of color are not able to access abortion if they must travel great distances to obtain abortion care [3].

Women having a second trimester or later abortion or having their procedure at a hospital were also more likely to travel greater distances because there are fewer of these providers. This finding is consistent with national data finding women travel greater distances for later abortions [3].

Traveling longer distances for care poses challenges beyond extra time in transit, particularly for low income women. Increased travel distance means increased costs for gas or public transit fare, hotel, loss of wages from time off work, and childcare, even though the actual procedure may be covered by public health insurance (as in California) or by abortion funds [47]. These costs can be compounded for low-income women, who may be less likely than wealthier women to own cars or have the flexibility to take time off work. Beyond costs, having to seek care outside of one’s community can add stress by isolating women from familiar surroundings and removing them from potential social support [48]. Women who would need to travel long distances to reach a Medi-Cal provider may instead opt to pay out-of-pocket for a closer provider, eschewing the benefit of Medicaid coverage. If the travel distance, costs, and other barriers are insurmountable, some women carry their unwanted pregnancies to term [19].

These findings highlight the need to pursue strategies to increase the number and geographic distribution of providers who accept Medi-Cal. Integrating early abortion into primary care settings has been shown to increase access without reducing safety or efficacy, [49, 50] and studies of women’s preferences suggest the majority of women would prefer to obtain an abortion at their primary care provider [51–54]. Increasing the types of providers who are qualified to provide abortion to include those more likely to work in rural and community-health settings than physicians can also increases access [55]. In California, legislation was passed in 2002 allowing nurse practitioners and nurse midwives to administer medication abortion without physician supervision [56] and in 2013 allowing them to conduct first trimester aspiration abortions without physician supervision [57]. Telemedicine programs, where a physician provides medication abortion by providing counseling via videoconference and then releases the medication via remote control, can also be a useful strategy to improve access where abortion providers are scarce [58].

Finally, efforts to increase the number of providers accepting Medi-Cal as payment for abortion suggest that reimbursement rates for abortion must be reevaluated. National studies have found that Medicaid abortion reimbursement levels are lower than insurance payments and also lower than what women paying out of pocket are charged [28]. In California, the second trimester abortion reimbursement rate by Medi-Cal FFS was substantially lower than private insurance when examined in 2006, less than half that of private insurance in some cases [29]. Reimbursement rates have not changed substantially since then (personal communication, Janley Hsiao, Billing Manager, Women’s Options Center). Increasing the rate of reimbursement, as well as making abortion coverage information clear and available and training Medi-Cal staff on abortion coverage, could potentially increase the number and distribution of providers accepting Medi-Cal.

This study has several limitations. First, this study relies on claims data which may contain erroneous codes [59]. Secondly, the location of the abortion provider may be inaccurate. While we aimed to ensure that we captured the location of abortion provision by use of billing address rather than other addresses, it is possible that the facility used a different administrative address than the location where the abortions occurred, reducing the accuracy of our distance calculation. Third, it is likely that women with Medi-Cal coverage paid out of pocket for abortion care due to difficulty getting Medi-Cal payment, to protect their privacy, or to obtain their abortion from a provider who did not accept Medi-Cal (for reasons of geographic proximity, trust, or a lack of knowledge about Medi-Cal abortion coverage), leading us to have underestimated the total number of abortions in this population. Furthermore, the study did not include women on other Medi-Cal plans including Presumptive Eligibility for Pregnant Women and Medi-Cal Access Program Medi-Cal plans, which are likely composed of younger women living in more urban areas and who would travel shorter distances to a provider. Finally, our data included only reimbursed claims, not all billed claims. It is possible that providers billed for abortions which were not reimbursed due to provider or Medi-Cal error. This could result in underestimation of both abortions and Medi-Cal FFS providers.

## Conclusions

In examining the distances that low-income women in California travel for Medicaid-covered abortion care, some women travel substantial distances despite relatively high accessibility in the state. Rural women travel particularly far despite having abortion rates comparable to their urban counterparts, highlighting the need for greater geographic distribution of abortion providers who accept Medicaid. This could be accomplished by increasing the Medi-Cal reimbursement rate, increasing the types of providers who provide abortions (including nurse practitioners, certified nurse midwives, and physician assistants) and expanding use of telemedicine among Medi-Cal providers. If national trends in declining unintended pregnancy and declining abortion rates continue, careful attention should be paid to ensure that reduced demand does not lead to greater disparities in geographic and financial access to abortion care by ensuring that providers accepting Medicaid payment are available and widely distributed.

## Additional file

Additional file 1: Additional description of data preparation. (DOCX 14 kb)

## Abbreviations

AOR: Adjusted odds ratio
API: Application programming interface
CPT: Current Procedural Terminology
DHCS: Department of Health Care Services
FFS: Fee-for-service
HCPCS: Healthcare Common Procedure Coding System
ICD-9: International Classification of Diseases 9th Revision
LMP: Last menstrual period
