# Supplementary material for: Distance traveled for Medicaid-covered abortion care in California
Source: BMC Health Serv Res. 2017 Apr 19;17:287. doi: 10.1186/s12913-017-2241-0 (PMC5395766; doi:10.1186/s12913-017-2241-0)
Supplement: Additional file 1: — Additional description of data preparation. (DOCX 14 kb) [file 12913_2017_2241_MOESM1_ESM.docx]

**Additional information on data preparation**

Abortions were identified using Healthcare Common Procedure Coding System (HCPCS) codes (59840–59841, 59850–59852 and 59855–59857, S0199, X7724, Z0336); Additionally, in some cases abortions were identified using a combination of both a “legal abortion” ICD-9 code (635) and either a treatment of miscarriage Current Procedural Terminology (CPT) code (59812, 59820, 59821) or a labor and delivery CPT code for inductions (0720, 0721, 59409). HCPCS and CPT codes also indicated the abortion type: 1) medication abortions, which includes use of mifepristone and misoprostol; 2) first trimester aspiration, which includes both manual and electric aspiration abortions as well as dilation and curettage “in the first 12–14 weeks of gestation” [1]; and 3) second-trimester or later procedures. We identified an abortion and any care up to 6 weeks after an abortion procedure; if another abortion procedure occurred within the 6 weeks, this was not considered a new abortion, but rather part of the care for the initial abortion. These methods were developed previously for an analysis on abortion safety [2].

We defined location of abortion care based on all claims on the day of the abortion; if abortion-related outpatient clinic or hospital claims appeared on the date of the abortion procedure together with claims from a physician’s office, the abortion was classified as taking place at the outpatient clinic or hospital. This was done because a physician may bill separately for their time, and that claim would not reflect the actual location of care. Women’s residence was categorized as urban or rural using the Rural Urban Commuting Area code for their zip code [3].

1. California Department of Health Care Services: **Medi-Cal Provider Manual**. In*.* Sacramento, CA: Department of Health Care Services; 2015.

2. Upadhyay UD, Desai S, Zlidar V, Weitz TA, Grossman D, Anderson P, Taylor D: **Incidence of emergency department visits and complications after abortion**. *Obstet Gynecol* 2015, **125**(1):175-183.

3. **Rural-urban commuting area codes** [<http://depts.washington.edu/uwruca/>]
